# Multidimensional approach to perceptions in football academies about educational, relational, inclusive, safety and social aspects among players, parents and coaches

**DOI:** 10.3389/fpsyg.2026.1906964

**Published:** 2026-10-06

**Authors:** Manuele Taleb, Giuseppe Giardullo, Ruggero Andrisano Ruggieri, Giuseppe Di Lascio, Pompilio Cusano, Felice Di Domenico

**Affiliations:** 1 Research Center of Physical Education and Exercise, University of Pegaso, Naples, Italy; 2 Department of Neuroscience, Biomedicine and Movement, University of Verona, Verona, Italy; 3 Department of Human, Philosophical and Education Sciences, University of Salerno, Fisciano, Italy; 4 Department of Educational and Sport Sciences, Pegaso University, Naples, Italy

**Keywords:** accountability, football academies, role-specific questionnaires, multi-stakeholder perceptions, young football players, youth sport, safeguarding, youth development

## Abstract

**Introduction:**

Youth football represents an important educational and social environment for young people’s development. Previous studies have typically assessed players, coaches, and parents using the same questionnaire to directly compare perceptions. Although methodologically rigorous, this approach may overlook role-specific perspectives. Therefore, this study aimed to analyse and compare the perceptions of players, coaches, and parents regarding the educational and social value of youth football from their respective viewpoints.

**Methods:**

A multidimensional, multi-stakeholder approach was adopted through a comparative re-analysis of data collected using three role-specific questionnaires administered to players, coaches, and parents. While differing in wording, the questionnaires addressed equivalent concepts across five contextual dimensions: (1) educational value of the football academy, (2) educational and communicative relationships, (3) inclusion and equity, (4) safety and relational climate, and (5) social function of the club. Composite indices were calculated for each dimension and analysed using descriptive statistics and between-group comparisons through Welch’s ANOVA.

**Results:**

No statistically significant differences emerged among players, coaches, and parents regarding the educational value of football academies, educational relationships, and inclusion-related dimensions. However, significant differences emerged in perceptions of safety and the club’s social function (*p* < 0.05). Coaches reported significantly higher perceptions of safety compared with the other stakeholder groups, whereas parents attributed greater social value to football academies than players did. Descriptive analyses further highlighted greater variability in players’ responses, suggesting that young athletes’ experiences are less homogeneous than the representations provided by adults.

**Discussion:**

These findings emphasize the importance of considering stakeholder-specific perspectives when evaluating the educational and social role of youth football. Greater alignment between organizational practices, educational objectives, and young athletes’ lived experiences may enhance the effectiveness of youth sport environments. Furthermore, the role-specific methodology adopted in this study generated stakeholder-relevant findings while preserving methodological rigor, offering a valuable framework for future research in youth sport settings.

## 1 Introduction

Youth football is now one of the main educational, social and relational settings frequented by children and adolescents outside the school environment. Within the Italian football system, football academies can be understood as organized youth football structures within sports clubs that provide a continuous developmental pathway across age-based categories, combining regular training and participation in competitive activities with educational, safeguarding and player-care functions aimed at supporting the broader development and well-being of young players (Federazione Italiana Giuoco Calcio – Settore Giovanile e Scolastico (FIGC-SGS), 2026). These academies do not merely fulfill a technical and sporting role focused on performance, but are increasingly taking on a broader educational responsibility, influencing processes of socialization, personal development, psychological well-being, inclusion and community participation. From this perspective, the youth sports context can be interpreted as a genuine educational agency complementary to school and the family, capable of influencing the development of transferable skills, social relationships and behavioral models (Raiola et al., 2023). From a pedagogical perspective, sport can represent a cross-curricular educational context capable of integrating motor learning, relational development and social participation (Altavilla et al., 2022). The educational value of sport should not be considered an automatic consequence of participation in sport but depends heavily on the pedagogical quality of the environment, the relational approaches adopted by adults and the organizational structure of the activities offered (Coakley, 2011). At the same time, various pedagogical approaches applied to sport have demonstrated significant effects on the development of awareness, responsibility, cooperation and social skills among young participants (Raiola et al., 2022; D’Isanto et al., 2022a). A further line of research has also highlighted how ecological-dynamic and constraints-led methodologies can promote active participation, adaptive decision-making and educational engagement among young athletes (Esposito et al., 2024). Sporting activity has also been associated with well-being, educational participation, social growth and the development of physical literacy in young participants (Raiola, 2025a).

Within football academies, the educational process simultaneously involves different stakeholders, each with different expectations, perceptions and responsibilities. Parents, coaches and young athletes experience the sporting context through complementary but not necessarily coinciding perspectives. Coaches tend to focus more on organizational, methodological and training aspects; parents often view the club as a space for educational development and social protection; young athletes experience first-hand the emotional, relational and identity-forming dynamics of daily sporting practice. From this perspective, educational perceptions have also been investigated in informal sporting contexts and football academies (Ceruso et al., 2020). Recent scientific studies have shown that sporting contexts can foster the development of game intelligence, decision-making skills, and non-verbal and interpersonal communication processes (Esposito et al., 2020a). Furthermore, the educational relationship and the quality of communication play an important role in the processes of motivation and participation in youth sport (Jowett and Ntoumanis, 2004). For this reason, a multidimensional analysis of the perceptions of various stakeholders represents a particularly relevant approach to understanding the actual educational and social quality of football academies. Multidimensional and multi-stakeholder approaches have received increasing attention in sporting and educational contexts. Other authors have investigated the perceptions of different actors regarding training, inclusive and pedagogical processes in sport and physical education (Di Domenico et al., 2024). In parallel, inclusion, educational communication and the relational climate have been identified as relevant to youth sports participation processes (Aliberti et al., 2022). Furthermore, some studies have emphasized the educational value of rules, cooperation and relational dynamics in youth sports contexts (Esposito et al., 2020b). Additionally, sport may also foster a sense of belonging at school, educational participation and social development among young participants (D’Elia, 2019).

However, despite the growing focus on the educational and social dimensions of youth sport, there is still limited scientific evidence that simultaneously compares the perceptions, opinions and awareness of parents, coaches and players within an integrated multidimensional framework. Within this line of research, a further aspect that has yet to be thoroughly explored concerns the ways in which such comparisons are methodologically constructed. In most comparative studies, the comparison between different stakeholders is carried out by administering the same assessment tool to the various groups involved, implicitly assuming that the same educational and social dimensions are perceived through equivalent interpretative categories. Although this approach facilitates the direct comparability of the data collected, it may limit the ability to capture perceptual elements closely linked to the specific nature of the role held within the sports system. Parents, coaches and young athletes view the football environment through the prism of differing responsibilities, expectations, experiences and levels of involvement, suggesting that, alongside comparisons between groups, greater attention should be paid to the specific perspectives of each stakeholder, whilst maintaining a shared conceptual framework (Raiola, 2025b). In particular, the literature still appears to be lacking in terms of an interconnected analysis of the educational, relational, inclusive, safety and social dimensions within football academies, and in terms of the development of methodological models capable of comparing different stakeholders without sacrificing the specific perspectives of everyone. This absence limits our understanding of any perceptual convergences or divergences among the stakeholders involved and reduces the possibility of developing models of educational accountability that are truly participatory and methodologically sensitive to the characteristics of the different actors involved.

The aim of this study was to conduct a comparative analysis of the perceptions of parents, coaches and young footballers regarding the educational, relational, inclusive, safety and social dimensions of youth football academies, using a multidimensional, multi-stakeholder approach designed to preserve the specific perspectives of the various stakeholders whilst ensuring that these remain comparable.

## 2 Materials and methods

### 2.1 Study design and participants

This study takes the form of a secondary analysis of data collected via questionnaires previously administered to three different groups of stakeholders involved in youth football: parents, coaches and players. No new questionnaires were administered for the present analysis. Instead, the previously collected responses were re-analysed by identifying conceptually corresponding items across the three role-specific questionnaires and aggregating them into five shared composite dimensions to enable between-group comparisons.

The sample of coaches consists of six individuals, with professional experience ranging from 3 to 12 years. Most of the coaches hold a FIGC Level 3 qualification, whilst some report having undergone informal or partial training. On average, the coaches devote between 12 and 34 h per week to coaching and oversee groups of between 8 and 25 players from different age groups. All the coaches involved report having taken part in at least one coaching course in the last year. The sample of footballers consists of 30 male athletes, aged between 10 and 17, belonging to Under-12/Under-13 (Esordienti) and Under-16/Under-17 (Allievi) age categories. The participants play various positions (goalkeeper, defender, midfielder, forward), with between 2 and 7 years’ experience in the sport. The weekly training frequency ranges from 2 to 5 sessions, and most athletes regularly take part in training sessions and official competitions (championships and tournaments). The group of parents consists of 20 participants, aged between 28 and 65, comprising 11 mothers and 9 fathers. The parents have children aged between 10 and 17, who have been members of the sports club for between 2 and 7 years. The sample includes parents with varying lengths of membership and levels of involvement in the club’s activities. During the initial data collection phase, participants were asked to provide informed consent to take part, with explicit indication of the survey’s research objectives and the use of data exclusively for research purposes. The data used in this analysis were processed in a completely anonymous form; the questionnaires contained no identifying information, and the responses were analysed exclusively in aggregate form. The data were used in accordance with the ethical principles of research in the educational and social fields, ensuring the protection of participants’ confidentiality. The original questionnaire-based data collection was approved by the Ethics Committee of Pegaso University (Prot./E 004726, 15 July 2025).

### 2.2 Data collection

The study utilizes three different questionnaires, separately administered to parents, coaches, and players, designed to investigate, from their respective perspectives, the same general construct: the educational value of youth football. Although the questionnaires feature wording and structures adapted to the participants’ roles, they share a common core theme, aimed at exploring the educational, relational, inclusive, safety and social aspects of the sporting context. Using these tools, a comparative conceptual analysis of the items was conducted with the aim of identifying overlapping content areas across the three questionnaires and reconstructing common thematic matrices that would allow for a comparison of the perceptions of the various stakeholders. In order to make the responses of the three groups comparable, a conceptual analysis of the items in the three questionnaires was carried out to identify cross-cutting dimensions. For each dimension, conceptually equivalent items were selected from the three questionnaires and subsequently aggregated into composite indices calculated as the arithmetic mean of the corresponding item scores. The five dimensions identified are as follows:

1. The educational value of football (EDU) examines the role of football as a context for personal growth, the development of responsibility and social-emotional skills, and the prevention of risky behavior.
2. Relationships and educational communication (REL) focuses on the quality of educational communication, active listening and active engagement.
3. Inclusion and Equity (INC) explores the themes of equal access, non-discrimination, and social and cultural inclusion.
4. Safety and Relational Climate (SAFE) concerns the perception of physical, emotional and relational safety, conflict management and the prevention of risky behavior.
5. Social Function of the Club (SOC) analyses the role of the club as a social and local actor, capable of fostering a sense of belonging and positive relationships.

Table 1 lists the items selected and aggregated for each conceptual dimension across the three stakeholder groups, highlighting the thematic correspondences used to construct the comparative composite indices.

**TABLE 1:** Conceptual correspondence of items across stakeholder groups and construction of multidimensional composite indices.

Item	Parents	Coaches	Players
EDU	– Activities promote autonomy and responsibility. – Effects on academic performance and motivation. – Reduction of risky behaviors.	– Activities programme develops life skills. – Involvement of players in decision-making. – Management of emotions and conflicts.	– Activities promote respect for rules and responsibility. – I feel that sport improves my wellbeing outside the playing field.
REL	– Communication between the club and families. – Family participation. – I feel listened to.	– Individualized formative feedback. – Collaboration with families and services.	– I feel involved in activities. – I feel listened to when I make suggestions or experience difficulties. – I receive clear and useful feedback from my coach.
INC	– The club promotes inclusion. – Discrimination/favoritism (reverse).	– Equal opportunities (playing time). – A club culture oriented toward educational objectives. – Training on inclusion.	– The club is inclusive toward people from different cultures. – Playing time is allocated fairly. – I feel isolated (reverse). – I have witnessed insults or discrimination (reverse).
SAFE	– Safety and safeguarding. – Management of conflicts and bullying.	– I do not have channels available to report concerns (reverse).	– I feel safe on the pitch and in the changing room. – The environment promotes respect and fair play. – The environment reduces risky behaviors.
SOC	– Collaboration with schools. – Collaboration with the local community. – Value for the local community.	– Collaboration with families and services. – Social impact within the local community. – Educational objectives of the club.	– The club organizes initiatives with schools and the local community. – The club makes me feel part of a community. – I have made new friendships here.

### 2.3 Statistical analysis

Based on the original questionnaires, a preliminary recoding of the selected items was carried out, including the reversal of scores for negatively worded items. For each participant, five composite indices (EDU, REL, INC, SAFE, and SOC) were calculated as the arithmetic mean of the items belonging to the same conceptual dimension. Descriptive statistics (mean and standard deviation) were calculated for each index separately for parents, coaches, and players. The distributional characteristics of the composite indices were examined using normality tests, including the Shapiro–Wilk test, while homogeneity of variances across stakeholder groups was assessed using Levene’s test. The three stakeholder groups were unequal in size (parents, *n* = 20; coaches, *n* = 6; players, *n* = 30), and evidence of variance heterogeneity was observed for some dimensions. Therefore, Welch’s ANOVA was adopted as the primary omnibus procedure across all five dimensions because of its greater robustness to unequal variances and unequal group sizes. For each Welch ANOVA, an omnibus effect size was estimated using weighted eta-squared (η^2^w), together with its 95% confidence interval, following the approach proposed for one-way heteroscedastic ANOVA models. When the omnibus Welch test was statistically significant, pairwise comparisons were performed using Games-Howell, which does not require equal variances or equal sample sizes. For these comparisons, mean differences, Games-Howell adjusted *p*-values, and 95% confidence intervals were reported. Cohen’s *d* with 95% confidence intervals was provided as a standardized pairwise effect-size measure (d ≈ 0.2 = small, d ≈ 0.5 = medium, *d* ≥ 0.8 = large). Data analysis was conducted using IBM SPSS Statistics software (version 26.0). Weighted eta-squared effect sizes and their 95% confidence intervals were calculated using Python according to the procedure described by Shieh (2013).

## 3 Results

Table 2 summarizes the descriptive statistics for the five indices for each stakeholder group. Mean scores were high across dimensions, although variability differed across groups and indices. A greater dispersion was observed for the INC among players and SOC among coaches.

**TABLE 2:** Descriptive statistics of multidimensional indices across stakeholder groups.

Item	Parents		Coaches		Players	
	M	SD	M	SD	M	SD
EDU	4.38	0.41	4.17	0.34	4.12	0.41
REL	3.94	0.23	3.92	0.20	3.88	0.25
INC	3.90	0.53	3.73	0.26	3.88	0.69
SAFE	4.03	0.26	4.83	0.41	4.23	0.41
SOC	4.07	0.34	3.83	0.75	3.60	0.41

M, mean; SD, standard deviation.

Given the unequal group sizes (parents, *n* = 20; coaches, *n* = 6; players, *n* = 30) and evidence of variance heterogeneity for some dimensions (Levene’s *p* = 0.030 for INC and *p* < 0.001 for SAFE), Welch’s ANOVA was used as the robust omnibus test. Welch’s ANOVA revealed no statistically significant between-group differences for the educational value index, Welch’s *F*(2, 15.05) = 2.43, *p* = 0.122, η^2^w = 0.083, the relational index, Welch’s *F*(2, 15.16) = 0.37, *p* = 0.699, η^2^w = 0.013, or the inclusion and equity index, Welch’s *F*(2, 23.40) = 0.67, *p* = 0.523, η^2^w = 0.024. A statistically significant between-group difference emerged for SAFE, Welch’s *F*(2, 13.52) = 10.80, *p* = 0.002, η^2^w = 0.288, and for SOC, Welch’s *F*(2, 12.61) = 9.09, *p* = 0.004, η^2^w = 0.255. A detailed description is shown in Table 3.

**TABLE 3:** Welch ANOVA results for multidimensional indices across stakeholder groups.

Item	Welch’s *F*	df1	df2	*p*	η^2^w	95% CI
EDU	2.43	2	15.05	0.122	0.083	[0.000, 0.233]
REL	0.37	2	15.16	0.699	0.013	[0.000, 0.097]
INC	0.67	2	23.40	0.523	0.024	[0.000, 0.126]
SAFE	10.80	2	13.52	**0.002**	0.288	[0.053, 0.476]
SOC	9.09	2	12.61	**0.004**	0.255	[0.034, 0.444]

Welch’s *F* = robust Welch analysis of variance statistic; df1 = numerator degrees of freedom; df2 = denominator degrees of freedom; *p* = significance level; η^2^w = weighted eta-squared effect size for the heteroscedastic one-way design; CI = confidence interval. Boldface is used to highlight statistically significant *p*-values (*p* < 0.05).

Games–Howell post-hoc comparisons showed that coaches reported higher SAFE scores than parents (mean difference = 0.808, *p* = 0.008, Cohen’s *d* = 2.75) and players (mean difference = 0.607, *p* = 0.029, Cohen’s *d* = 1.49). The difference between parents and players was not statistically significant (mean difference = −0.202, *p* = 0.091). Parents attributed greater social value to the club than players did (mean difference = 0.467, *p* < 0.001, Cohen’s *d* = 1.21). No statistically significant differences were observed between parents and coaches (*p* = 0.747) or between coaches and players (*p* = 0.758). A detailed description is shown in Table 4.

**TABLE 4:** Games–Howell *post-hoc* comparisons for SAFE and SOC.

Item	Comparison	Mean difference	95% CI	*p*	Cohen’s d	95% CI for d
SAFE	Parents-coaches	−0.808	[−1.343, −0.273]	**0.008**	−2.75	[−3.93, −1.54]
	Parents-players	−0.202	[−0.429, 0.025]	0.091	−0.57	[−1.14, 0.01]
	Coaches- players	0.607	[0.072, 1.142]	**0.029**	1.49	[0.53, 2.42]
SOC	Parents- coaches	0.237	[−0.754, 1.227]	0.747	0.52	[−0.41, 1.44]
	Parents- players	0.467	[0.208, 0.725]	**<0.001**	1.21	[0.59, 1.82]
	Coaches- players	0.230	[−0.760, 1.220]	0.758	0.48	[−0.41, 1.36]

Positive mean differences indicate higher values for the first group listed; *p*-values are Games–Howell adjusted; Cohen’s d were interpreted as small (0.2), medium (0.5), and large (≥0.8); CI = confidence interval. Boldface is used to highlight statistically significant *p*-values (*p* < 0.05).

## 4 Discussion

The results of this study revealed no statistically significant differences among parents, coaches and young footballers regarding the educational, relational and inclusive dimensions, whilst statistically significant differences emerged in the dimensions of safety and the club’s social function. These results suggest that football academies are generally perceived as positive educational environments; however, differences in perception persist in the dimensions most closely linked to subjective experience, relational protection and the social significance attributed to the club.

With regard to the educational dimension, no statistically significant differences emerged between stakeholders (Welch’s *F*(2, 15.05) = 2.43, *p* = 0.122). This result indicates that no statistically significant differences in the perceived educational role of football academies were detected among parents, coaches and players. The high mean scores observed across the three groups seem to indicate that participation in football is widely perceived as capable of promoting responsibility, autonomy, personal growth and psychosocial well-being. These findings are consistent with the Positive Youth Development framework, according to which sport can foster personal and social skills when embedded in supportive educational environments (Holt and Neely, 2011; Hambali et al., 2022). At the same time, various educational studies have shown how heuristic and educational approaches can positively influence awareness, collaboration and responsibility among young athletes (D’Isanto et al., 2022b). Further contributions relating to ecological-dynamic and constraints-led approaches have also highlighted how active participation and decision-making processes can foster empowerment and educational engagement in young athletes (D’Isanto et al., 2021). However, some authors emphasize that the educational effects of sport depend heavily on the pedagogical quality of the environment and not solely on sporting participation.

No statistically significant differences emerged in the relational dimension either (Welch’s *F*(2, 15.16) = 0.37, *p* = 0.699), suggesting that no between-group differences in communication and listening processes emerged among the different stakeholder groups. This finding appears consistent with the literature highlighting the central role of educational communication and the coach-athlete relationship in the motivational and participatory processes of youth sport. Similarly, the role of non-verbal and relational communication in educational sporting processes has also been highlighted in previous pedagogical studies. Simultaneously, international literature highlights how environments that are overly performance-oriented can reduce young athletes’ perception of being listened to and actively involved (Appleton and Duda, 2016).

Regarding the inclusive dimension, no statistically significant differences emerged (Welch *F*(2,23.40) = 0.67, *p* = 0.523). However, the greater variability observed in the responses of young footballers suggests that inclusion may be experienced in a less uniform manner than perceived by adults. This element appears particularly relevant as it highlights how inclusion may represent a formally shared value but one that is concretely influenced by peer relationships and the daily experiences of athletes. Previous studies have emphasized that inclusion in sport requires not only declarative educational principles, but also organizational and relational practices concretely oriented toward participatory equity and social recognition.

The most significant differences emerged in the area of safety, where a statistically significant difference was observed between stakeholders (Welch *F*(2, 13.52) = 10.80, *p* = 0.002). Post-hoc comparisons showed that coaches reported significantly higher levels of perceived safety than parents (*d* = 2.75) and players (*d* = 1.49). This finding suggests that there is a discrepancy in perception between those who organize and manage the sporting environment and those who experience it on a daily basis. Coaches may interpret safety primarily in organizational and procedural terms, whilst young athletes appear to experience it through emotional, relational and psychological dimensions as well. These findings are consistent with the international Safe Sport framework, according to which safety in sport cannot be limited solely to physical protection, but must also include emotional well-being, mutual respect and the prevention of harmful behavior (Kerr and Stirling, 2012). Furthermore, previous studies have shown that adults frequently tend to overestimate the quality and safety of sporting environments compared to the athletes’ own perceptions (Furusa et al., 2024).

The social dimension also revealed statistically significant differences (Welch *F*(2, 12.61) = 9.09, *p* = 0.004), with parents attributing significantly greater social value to the club than young footballers (*d* = 1.21). This finding suggests that parents tend to view the club as a broader educational and community institution, capable of fostering a sense of belonging and positive social development, whilst young athletes appear to experience the football environment primarily through their immediate sporting and social interactions. These findings are consistent with the literature that interprets sports organizations as potential generators of social capital and community cohesion (Coalter, 2007). At the same time, previous studies on educational accountability in football have highlighted that football academies can only be regarded as public goods when their social function is genuinely recognized and shared by the various stakeholders.

Overall, the five dimensions considered should not be interpreted as isolated components, but as deeply interconnected dimensions that simultaneously contribute to the overall educational quality of football academies. The absence of statistically significant differences in the educational, relational and inclusive dimensions appears, in fact, to indicate a relatively similar pattern across stakeholders, while the divergences that emerged in the dimensions of safety and social function highlight how stakeholders’ subjective experiences may differ when aspects more closely linked to emotional protection, the perception of the relational climate and the social significance attributed to the club are involved. From this perspective, the present study reinforces the idea that the educational value of sport does not automatically emerge from mere participation, but depends on the continuous interaction between organizational practices, relational processes, pedagogical intentions and the experiential lives of young athletes. These findings also confirm the complexity of the perceptual dynamics at play in youth sports contexts and the importance of taking into account the experiences of young athletes, parents and sports organizations simultaneously when creating genuinely participatory educational environments (Furusa et al., 2021).

### 4.1 Limitations of the study

The main limitations of this study include the small sample size, particularly within the group of coaches, the exploratory and cross-sectional nature of the research design, and the use of self-report questionnaires that may be subject to social desirability bias. Furthermore, the player sample consisted exclusively of male athletes, and the study was conducted within a limited geographical context. The available demographic information did not allow specific comparisons according to sex/gender or sociocultural background. These characteristics should be considered when interpreting the generalizability of the findings and should be addressed in more heterogeneous, multicenter samples. Consequently, the results should be interpreted with caution and cannot be generalized to the entire population of youth football academies.

### 4.2 Practical implications

From a practical perspective, the findings suggest the importance of developing multi-stakeholder monitoring systems within football academies, directly involving players, parents and coaches in educational and organizational evaluation processes. At the same time, safeguarding and relational safety strategies should be further strengthened through more participatory and visible systems of listening, prevention and support for young players. Furthermore, the social role of football academies should be made more explicit and genuinely participatory for the young people themselves, so that the social value attributed by adults can be truly experienced and perceived by the young participants. Feedback arising from multidimensional dialogue between stakeholders could also serve as a useful tool for implementing and improving the organizational and educational structure of informal learning contexts such as youth football academies. From this perspective, the integration of educational, relational, social, inclusive and safety dimensions could contribute not only to improving the sporting experience, but also to generating positive effects on the wider social and educational fabric surrounding young athletes, thereby strengthening the educational and social accountability of football academies within local communities. Building on these findings, the multidimensional approach may inform a practical three-axis educational framework involving parents, coaches, and players. The parent axis would emphasize constructive involvement, dialogue, and participation in the educational process; the coach axis would focus on communication, listening, individualized relational support, and responsible leadership; and the player axis would promote active participation, communication with significant adults, and access to relational and educational support within the football academy. This framework should be considered a practice-oriented proposal derived from the present findings rather than a validated intervention, and its feasibility and effectiveness should therefore be prospectively evaluated in youth football settings.

### 4.3 Future perspectives

Future research could expand this multidimensional framework through larger, multicenter samples, incorporating mixed methods approaches to gain a deeper understanding of stakeholders’ differing perceptions. Specifically, quantitative comparisons of the five composite dimensions could be complemented by semi-structured interviews or focus groups with players, parents and coaches to explore the reasons underlying similarities and differences in their perceptions. Integrating these qualitative accounts with questionnaire findings could provide a more contextualized interpretation of stakeholder experiences within football academies. Future research could also investigate talent-development pathways, with particular attention to coach–parent interactions and to the balance between increasing sporting demands and young athletes’ educational commitments. Further longitudinal studies could also analyse how educational, relational and social perceptions evolve over time and whether experiences within stakeholder-aligned youth football environments are associated with broader developmental outcomes beyond the immediate sporting context. Cross-club and cross-cultural comparisons could examine whether the perceptions of players, parents and coaches differ across football academies in relation to differences in organizational culture and structure, as well as in the resources available and employed within each academy. Furthermore, future research could compare different age groups, competitive levels and sports disciplines in order to understand how educational, relational, inclusive, social and safety perceptions may vary across different organizational and training contexts in youth sport.

## 5 Conclusion

This study highlights how youth football academies can serve as meaningful educational and social environments only when the perceptions and experiences of the various stakeholders are continually considered, integrated and transformed into concrete processes of educational and organizational improvement. The absence of statistically significant differences in the educational, relational and inclusive dimensions suggests a relatively similar pattern of perceptions among parents, coaches and young athletes, whilst the divergences that emerged in the dimensions of safety and social function highlight the need for greater attention to be paid to the athletes’ own subjective experiences. The multidimensional, multi-stakeholder framework also helps to reinforce the concept of educational and social accountability within football academies, highlighting how such contexts can play a significant role not only in the sporting development of young players, but also in fostering processes of participation, social belonging, inclusion and relational well-being.

## Data Availability

The original contributions presented in this study are included in the article/supplementary material. The original datasets collected through the three role-specific questionnaires, prior to the comparative re-analysis conducted in this study, are available from the corresponding author upon reasonable request.
